# Notes on *Macroteleia* Westwood (Hymenoptera, Scelionidae) from China, with description of a new species

**DOI:** 10.3897/zookeys.939.51272

**Published:** 2020-06-09

**Authors:** Chun-Dan Hong, Ovidiu Alin Popovici, Hua-Yan Chen

**Affiliations:** 1 Bureau of Agriculture and Rural Affairs of Longhu, Shantou 515000, China Bureau of Agriculture and Rural Affairs of Longhu Shantou China; 2 University “Al. I. Cuza” Iași, Faculty of Biology, CERNESIM, Boulevard Carol I 11, RO-700506, Iași, Romania University “Al. I. Cuza” Iași Iași Romania; 3 State Key Laboratory of Biocontrol, School of Life Sciences / School of Ecology, Sun Yat-sen University, Guangzhou 510275, China Sun Yat-sen University Guangzhou China

**Keywords:** Egg parasitoid, new distribution record, Platygastroidea, redescription, taxonomy

## Abstract

The wasp genus *Macroteleia* Westwood from China has been previously revised, but some species are only known from males. Here the females of two known species are described: *M. carinigena* Chen, Johnson, Masner & Xu and *M. gracilis* Chen, Johnson, Masner & Xu. In addition, one species is redescribed: *M. variegata* Kozlov & Kononova; and one species is described as new: *Macroteleia xui* Hong & Chen, **sp. nov.***Macroteleia ischtvani* Kononova, **syn. nov.** is proposed as new synonym of *M. variegata* Kozlov & Kononova.

## Introduction

The species of the wasp genus *Macroteleia* Westwood are egg parasitoids of longhorned grasshoppers (Orthoptera, Tettigoniidae) (Muesebeck 1977). These wasps are spread worldwide, except Antarctica, but most species occur in tropical and subtropical regions (Masner 1976; Chen et al. 2013). Species of *Macroteleia* are easily recognized because of the unarmed propodeum, the marginal vein as long as, or longer, than the stigmal vein, and the peculiar shape of T6 in female (strongly compressed laterally) (Chen et al. 2013). The Chinese fauna of *Macroteleia* have been revised by Chen et al. (2013), with several new species described from the tropical and subtropical regions of China. However, of the seven new species proposed by Chen et al. (2013), three species were described based only on males. Considering the sexual dimorphism (displayed especially in the structure of the antenna and in the shape and the structure of the metasoma) and the importance of the shape of metascutellum and the structure of propodeum (divided, or not, into two lobes) in females to separate species of *Macroteleia* (Muesebeck 1977; Chen et al. 2013), the discovery of females in species known only from the males should enhance our knowledge of the concept of these species.

In this study the females of two species, previously known only from males, are described. Furthermore, a newly recorded species (*Macroteleia variegata* Kozlov & Kononova, 1987) from China is redescribed and another, *Macroteleia xui* is described as new for science.

## Materials and methods

This work is based upon specimens in the following collections, with abbreviations used in the text: **BMNH**, The Natural History Museum, London, UK; **IZCAS**, Institute of Zoology, Chinese Academy of Sciences, Beijing, China; **SCAU**, Hymenoptera Collection, South China Agricultural University, Guangzhou, China; **SYSBM**, Sun Yat-sen University, The Museum of Biology, Guangzhou, China; **UASK**, Schmalhausen Institute of Zoology of National Academy of Sciences of Ukraine, Kiev, Ukraine.

Abbreviations and morphological terms used in text: **A1, A2, ..., A12**: antennomere 1, 2, …, 12; **LOL**: lateral ocellar line, shortest distance between inner margins of median and lateral ocelli (Masner 1980); **OOL**: ocular ocellar line, shortest distance from inner orbit and outer margin of posterior ocellus (Masner 1980); **POL**: posterior ocellar line, shortest distance between inner margins of posterior ocelli (Masner 1980); **T1, T2, ..., T7**: metasomal tergite 1, 2, ..., 7; **S1, S2, …, S7**: metasomal sternite 1, 2, …, 7. Morphological terminology otherwise generally follows Masner (1980), Mikó et al. (2007) and Chen et al. (2013).

In the Material examined section the specimens studied are recorded in an abbreviated format, using unique identifiers (numbers prefixed with “SCAU”) for the individual specimens. The label data for all specimens have been georeferenced and recorded in the Hymenoptera Online database; details on the data associated with these specimens can be accessed at mbd-s.asc.ohio-state.edu by entering the identifier in the search form (note the space between the acronym and the number).

Images and measurements were made using Nikon SMZ25 microscope with a Nikon DS-Ri 2 digital camera system. Images were post-processed with Abobe Photoshop CS6 Extended.

## Taxonomy

### Macroteleia carinigena

Taxon classificationAnimaliaHymenopteraScelionidae

Chen, Johnson, Masner & Xu, 2013

EB4C926F-7477-5734-AE56-DEC1414C135B

http://zoobank.org/42427976-EE7B-4B81-8910-EF308AE8716E

Figures 1–6

Macroteleia carinigena Chen, Johnson, Masner & Xu, 2013: 13, 19 (original description, keyed).

#### Material examined.

***Holotype***, male: China: Hainan Prov., Mount Yinggeling, 28.V.2007, L.-Q. Weng, SCAU 000032 (deposited in SCAU). ***Paratypes***: China: 1 male, Hainan, Mt Diaoluo, 18°39'N, 109°53'E, 29.V.2007, Bin Xiao, SCAU 000033 (SCAU); 1 male, China: Hainan, Mt Diaoluo, 18°39'N, 109°53'E, 29.V.2007, Jingxian Liu, SCAU 000034 (SCAU).

#### Other material.

China: 2 females, Hainan, Mt Diaoluoshan, 18°39'N, 109°53'E, 16–17.VII.2006, Jingxian Liu, SCAU 3040365, 3040366, 3048585 (SYSBM).

**Figures 1–6.**
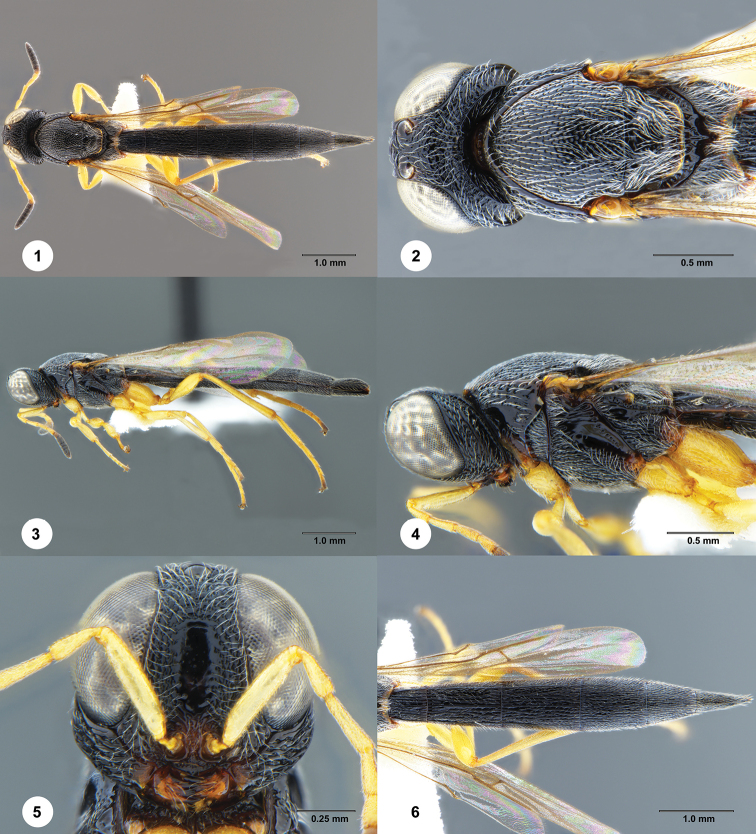
*Macroteleia carinigena* Chen, Johnson, Masner & Xu, female (SCAU 3048585) **1** dorsal habitus **2** head and mesosoma, dorsal view **3** lateral habitus **4** head and mesosoma, lateral view **5** head, anterior view **6** metasoma, dorsal view.

#### Description.

**Female.** Body length: 6.88–6.94 mm (*N* = 3).

***Color*.** Body black; mandible reddish brown; palpi yellow; legs yellow throughout; A1–A5 yellow, remainder of antenna dark brown to black; fore wing hyaline.

***Head*.** Transverse in dorsal view, 1.4–1.56 × as wide as long, slightly wider than mesosoma; lateral ocellus contiguous with inner orbit of compound eye; POL 1.5–1.67 × LOL; occipital carina continuous medially, irregularly crenulate throughout; central keel absent; medial frons punctate with irregularly shaped smooth area; ventrolateral frons punctate rugulose to densely punctate; frons below median ocellus punctate reticulate; vertex densely punctate with punctures in part contiguous; gena with a strong carina parallel to occipital carina, punctate rugose dorsally; length of A3 1.24–1.30 × length of A2.

***Mesosoma*.** Cervical pronotal area densely punctate; dorsal pronotal area areolate; lateral pronotal area smooth dorsally, irregularly depressed ventrally; netrion densely finely punctate; notaulus shallow, irregularly foveolate; middle lobe of mesoscutum densely punctate, becoming denser anteriorly and posteriorly; lateral lobes of mesoscutum densely punctate throughout; mesoscutellum densely punctate, becoming denser laterally; metascutellum transverse, posterior margin slightly pointed medially, longitudinally carinate; propodeum continuous medially, not divided into two separated lobes, posterior margin narrowly notched medially, each side with rugose sculpture covered by dense, recumbent, white setae; upper mesepisternum with a row of robust longitudinal carinae below subalar pit; lower mesepisternum densely punctate rugulose; mesopleural depression smooth; metapleuron longitudinally striate with coarse punctures in interstices, or longitudinally punctate rugose.

***Legs*.** Slender; hind femur weakly swollen, 4.00–4.55 × as long as its maximum width; hind tibia without spines over outer surface; hind basitarsus 7.67–9.00 × as long as its maximum width.

***Wings*.** Apex of fore wing extending from as far as basal of T5; R 1.46–1.60 × as long as r-rs, R1 1.95–2.43 × length of R.

***Metasoma*.** Posterior margin of transverse sulcus on T2 strongly convex; sublateral tergal carinae well developed on T1–T3, weakly developed on anterior half of T4; T1–T4 sparsely longitudinally striate medially, with delicate punctures in interstices, punctate rugulose laterally; T5–T6 densely longitudinally striate, with numerous delicate punctures in interstices; length of T3 1.28–1.4 × length of T6; T5 distinctly longer than wide; S2–S6 densely longitudinally striate, with delicate punctures in interstices; prominent longitudinal median carina present on S2–S5.

#### Distribution.

China (Hainan).

### Macroteleia emarginata

Taxon classificationAnimaliaHymenopteraScelionidae

Dodd, 1920

3E792A6A-226C-58BD-A796-E83995B83E94

http://zoobank.org/42427976-EE7B-4B81-8910-EF308AE8716E

Figures 7–12

Macroteleia emarginata Dodd, 1920: 326 (original description); Masner 1965: 82 (type information); Johnson 1992: 426 (cataloged, type information); Chen, Johnson, Masner and Xu 2013: 12, 14, 33 (description, keyed, distribution).

#### Material examined.

***Holotype***, female, Malaysia: Kuching [Quop, Oct. 1906], [P. Cameron Coll. 1914-110], [*Macroteleia flavipes* Cam. Type Borneo], [*Macroteleia emarginata* Dodd. ♀ Type], [Type 9.480] (deposited in BMNH).

#### Other material.

China: 2 females, 1 male, Yunnan, Xishuangbanna, Menghai, Bulangshan Village, 21°44.746'N, 100°26'E, 1610 m, Area D, grass, MT (Malaise trap), 20.VI–20.VII.2018, Li Ma, SCAU 3048682–3048684 (SYSBM); 2 females, Yunnan, Xishuangbanna, Menghai, Bulangshan Village, 21°44.746'N, 100°26'E, 1610 m, Area D, grass, MT (Malaise trap), 17.V–20.VI.2018, Li Ma, SCAU 3048685, 3048686 (SYSBM).

#### Distribution.

China (Fujian, Hunan, Guangdong, Hainan, Guizhou, Yunnan); Malaysia.

#### Comments.

Chen et al. (2013) recorded this species from the Oriental Region of China based upon the careful description provided by Alan Dodd in the original publication. Here, we provide the images of the holotype and additional records of this species from China. The specimens examined by Chen et al. (2013) and the ones we record here match well with the holotype.

**Figures 7–12.**
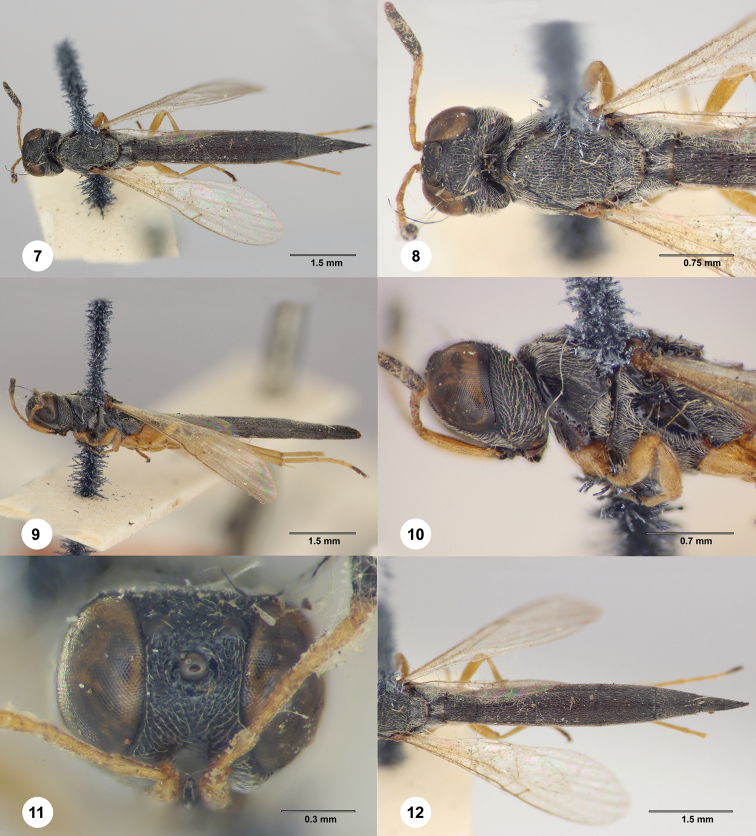
*Macroteleia emarginata* Dodd, holotype, female (B.M. TYPE HYM. 9.480) **7** dorsal habitus **8** head and mesosoma, dorsal view **9** lateral habitus **10** head and mesosoma, lateral view **11** head, anterior view **12** metasoma, dorsal view.

### Macroteleia gracilis

Taxon classificationAnimaliaHymenopteraScelionidae

Chen, Johnson, Masner & Xu, 2013

75A15EA7-A222-5796-9CCB-7E457D856AA3

http://zoobank.org/FC1AC5B9-9F13-4AC7-9057-7DD106F227AB

Figures 13–18

Macroteleia gracilis Chen, Johnson, Masner & Xu, 2013: 14, 40 (original description, keyed).

#### Material examined.

China: 1 female, Guangdong, Nanling Nature Reserve, 24°54'N, 113°00'E, 9–18.VII.2004, Juanjuan Ma, SCAU 3040368 (SYSBM); 1 male, Hainan, Haikou, Hainan University, Haidian campus, orchard, 20°3'15"N, 110°19'21"E, MT (Malaise trap), 14–20.IX.2017, Youxing Zhou, SCAU 3040367 (SYSBM); 1 male, Hainan, Haikou, Hainan University, Haidian campus, orchard, 20°3'15"N, 110°19'21"E, MT (Malaise trap), 3–9.VIII.2017, Youxing Zhou, SCAU 3040368 (SYSBM).

#### Description.

**Female.** Body length: 6.17 mm (*N* = 1).

***Color*.** Body black; mandible reddish brown; palpi yellow; legs yellow throughout; A1–A6 yellow, remainder of antenna dark brown to black; fore wing hyaline.

***Head*.** Transverse in dorsal view, 1.4–1.5 × as wide as long, slightly wider than mesosoma; lateral ocellus contiguous with inner orbit of compound eye; POL 1.5–1.54 × LOL; occipital carina continuous medially, irregularly punctate; central keel weakly developed, extending onto interantennal process; medial frons punctate rugose ventrally, with irregularly shaped smooth area dorsally; frons below median ocellus densely punctate; vertex sparsely punctate to smooth behind posterior ocelli, becoming densely punctate posteriorly; gena punctate rugose; length of A3 1.1–1.2 × length of A2.

***Mesosoma*.** Cervical pronotal area densely punctate; dorsal pronotal area punctate rugulose; lateral pronotal area smooth dorsally, punctate rugulose ventrally; netrion finely punctate rugulose; notaulus shallow, foveolate; middle lobe of mesoscutum densely punctate, sculpture becoming denser anteriorly; lateral lobes of mesoscutum densely finely punctate throughout; mesoscutellum densely finely punctate throughout; metascutellum transverse, posterior margin slightly pointed medially, longitudinally carinate; propodeum continuous medially, not divided into two separated lobes, posterior margin narrowly notched medially, each side with several irregular longitudinal carinae medially, otherwise punctate rugulose, covered by dense, recumbent, white setae; upper mesepisternum with a row of somewhat robust longitudinal carinae below subalar pit; lower mesepisternum variably smooth to punctate rugulose; mesopleural depression smooth; metapleuron longitudinally striate throughout.

***Legs*.** Slender; hind femur weakly swollen, 4.23–4.80 × as long as its maximum width; hind tibia without spines over outer surface; hind basitarsus 12.60–14.00 × as long as its maximum width.

***Wings*.** Apex of fore wing extending from as far as posterior margin of T4; R 2.06–2.46 × as long as r-rs, R1 1.63–1.90 × length of R.

***Metasoma*.** Posterior margin of transverse sulcus on T2 slightly convex; sublateral tergal carinae well developed on T1–T4, weakly developed on anterior half of T4; T1–T4 sparsely longitudinally striate medially, with delicate punctures in interstices, punctate rugulose laterally; T5–T6 densely longitudinally striate, with numerous delicate punctures in interstices; length of T3 0.90–0.95 × length of T6; T5 distinctly longer than wide; S2–S6 densely longitudinally striate, with delicate punctures in interstices; prominent longitudinal median carina present on S2–S4.

#### Distribution.

China (Guangdong, Hainan).

**Figures 13–18.**
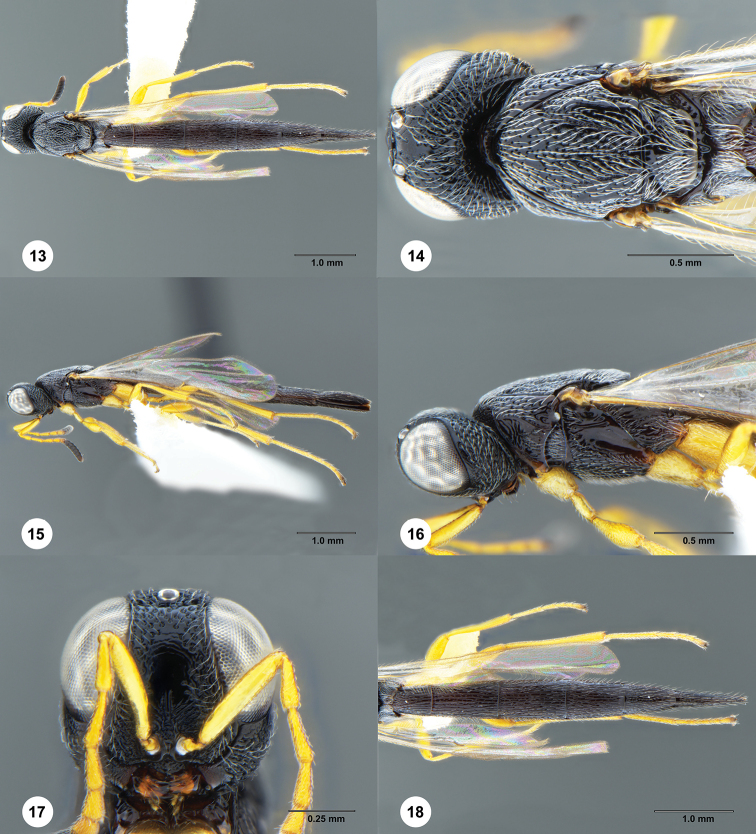
*Macroteleia gracilis* Chen, Johnson, Masner & Xu, female (SCAU 3048586) **13** dorsal habitus **14** head and mesosoma, dorsal view **15** lateral habitus **16** head and mesosoma, lateral view **17** head, anterior view **18** metasoma, dorsal view.

### Macroteleia xui

Taxon classificationAnimaliaHymenopteraScelionidae

Hong & Chen sp. nov.

314616C4-7295-5020-B49C-59A3A57824BD

http://zoobank.org/9A0F15EC-FD9A-4BC2-BB64-8053834F46C9

Figures 19–24

#### Material examined.

***Holotype***, female: China: Hebei, Baoding, Hebei Agricultural Unv., West Campus, MT, 38°49'44"N, 115°27'1"E, 30.VIII–6.IX.2017, Fan Fan, SACU 3040364 (deposited in SYSBM). ***Paratypes***: China: 1 female, Yunnan, Xishuangbanna, Menghai, Bulangshan Village, 1595 m, Area D, forest, 21°44.761'N, 100°25.959'E, 20.IV-20.VII.2018, MT (Malaise trap), Li Ma, SCAU 3040370 (SYSBM); 1 female, Shandong, Shanghe County, MT4, 37°16'4"N, 117°9'10"E, 18–24.VIII.2018, Jiahe Yan, SCAU 3048687 (SYSBM); 3 females, Shandong, Shanghe County, MT4, 37°16'4"N, 117°9'10"E, 7–14.IX.2018, Jiahe Yan, SCAU 3048593–3048595 (SYSBM).

#### Diagnosis.

This species is most similar to *M. striativentris* Crawford in color and size but can be distinguished by the medially divided propodeum and triangular metascutellum.

#### Description. Female.

Body length: 5.48–5.60 mm (*N* = 6).

***Color*.** Head and mesosoma black, metasoma dark brown to black; mandible brown with teeth dark brown; palpi yellow; legs pale brown throughout; A1–A6 yellow, remainder of antenna black; fore wing hyaline.

***Head*.** Transverse in dorsal view, 1.4–1.5 × as wide as long, slightly wider than mesosoma; OOL short, 0.17–0.20 × times minimum diameter of lateral ocellus; POL 1.5–1.54 × LOL; occipital carina continuous medially, irregularly punctate; central keel weakly developed, extending onto interantennal process; medial frons punctate rugose ventrally, with irregularly shaped smooth area dorsally; frons below median ocellus punctate rugulose; posterior vertex sparsely punctate rugulose behind posterior ocelli, becoming densely punctate posteriorly; gena punctate rugose; length of A3 as long as A2.

***Mesosoma*.** Cervical pronotal area densely punctate; dorsal pronotal area punctate rugulose; lateral pronotal area smooth dorsally, punctate rugulose ventrally; netrion densely finely punctate; notaulus shallow, foveolate; mesoscutum densely punctate; mesoscutellum moderately finely punctate throughout; metascutellum triangular, strongly produced medially, extending into space between propodeal lobes; propodeum narrowly divided into two subtriangular lobes, each side with several irregular longitudinal carinae medially, otherwise punctate rugulose; upper mesepisternum with a row of robust longitudinal carinae below subalar pit; lower mesepisternum variably smooth to punctate rugulose; mesopleural depression smooth; metapleuron longitudinally striate dorsally, punctate rugose ventrally.

***Legs*.** Slender; hind femur weakly swollen, 3.4–4.0 × as long as its maximum width; hind tibia without spines over outer surface; hind basitarsus 10.60–11.20 × as long as its maximum width.

***Wings*.** Apex of fore wing extending from as far as middle of T4; R 1.97–2.06 × as long as r-rs, R1 1.83–1.90 × length of R.

***Metasoma*.** Posterior margin of transverse sulcus on T2 straight; sublateral tergal carinae well developed on T1–T3; T1–T3 densely longitudinally striate medially, with delicate punctures in interstices, punctate rugulose laterally; T4–T6 densely longitudinally striate, with numerous delicate punctures in interstices; length of T3 0.78–0.81 × length of T6; T5 distinctly longer than wide; S2–S6 densely longitudinally striate, with delicate punctures in interstices; prominent longitudinal median carina present on S2–S4.

**Male.** Unknown.

#### Etymology.

This speices is named *xui* in honor of the late Professor Zaifu Xu for his great contribution to Chinese Hymenoptera taxonomy.

#### Distribution.

China (Hebei, Shandong, Yunnan).

**Figures 19–24.**
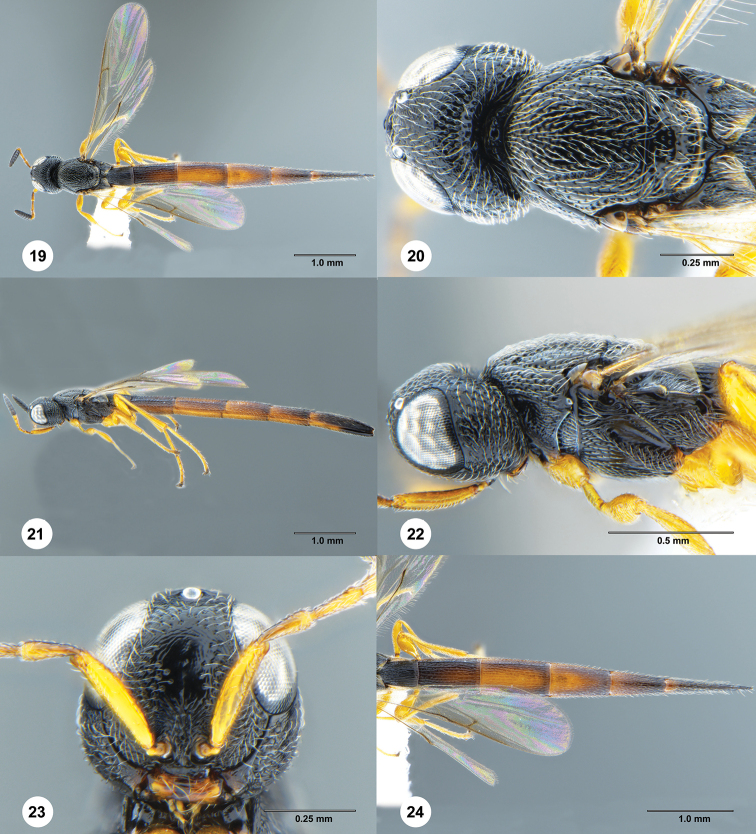
*Macroteleia xui* sp. nov., holotype, female (SACU 3040364) **19** dorsal habitus **20** head and mesosoma, dorsal view **21** lateral habitus **22** head and mesosoma, lateral view **23** head, anterior view **24** metasoma, dorsal view.

### Macroteleia variegata

Taxon classificationAnimaliaHymenopteraScelionidae

Kozlov & Kononova, 1987

5CF0095B-B5EA-581A-A6DC-1CD8ECDF1C13

http://zoobank.org/720C6A99-4641-4BAE-9B1D-39D3B55FB607

Figures 25–30 , 31–36

Macroteleia variegata Kozlov & Kononova, 1987: 94, 95, 99 (original description, keyed); Kozlov and Kononova 1990: 190, 199 (description, keyed); Johnson 1992: 433 (cataloged, type information); Kononova 1995: 70 (keyed); Kononova and Petrov 2003: 606 (keyed); Kononova and Kozlov 2008: 234, 248 (description, keyed).
Macroteleia ischtvani Kononova, 2008: 234, 250 (original description, keyed), syn. nov.

#### Material examined.

***Holotype***, female, *M. variegata*: Russia: [Primorskiy kr., Shkotovskiy r-n, okr. Apisimovki, Kononova 3.8.1977] [Holotypus *Macroteleia variegata*, Kononova], UASK 0104 (deposited in UASK). ***Holotype***, female, *M. ischtvani*: Hungary: [Hungary, Tiszaizolátum TIAD, 1995.08.15, leg. JATE ökológia] [Holotypus, *M. ischtvani*, Kononova], UASK 0100 (deposited in UASK).

#### Other material.

China: 1 male, Xinjiang, Gongliu County, Hetaogou, 43°25'38"N, 82°15'6"E, 1–2.VII.2016, Yicheng Li et al., yellow pan trap, SCAU 3048584 (SYSBM); 1 female, Hebei, Xiaowutai National Nature Reserve, 1364 m, 39°52.048'N, 114°56.446'E, 10–17.IX.2012, Malaise trap, Haiming Zhang, SCAU 3040369 (IZCAS); 1 female, Inner Mongolia, Xing’an Meng, 46°4'56"N, 122°2'15"E, 8.VIII.2011, Feng Yuan, SCAU 3041128 (IZCAS).

#### Redescription. Female.

Body length: 5.20–5.37 mm (*N* = 2).

***Color*.** Head yellow with upper frons and vertex dark brown to black; mesosoma variably yellow to dark brown; mandible yellow with teeth dark brown; palpi yellow; legs yellow throughout; A1–A5 brown, remainder of antenna dark brown to black; fore wing hyaline.

***Head*.** Transverse in dorsal view, 1.5–1.65 × as wide as long, as wide as mesosoma; OOL short, 0.20–0.30 × times minimum diameter of lateral ocellus; POL 1.38–1.4 × LOL; occipital carina interrupted medially; central keel weakly developed, extending onto interantennal process; medial frons punctate rugose ventrally, with irregularly shaped smooth area dorsally; frons below median ocellus densely punctate; posterior vertex rugulose behind posterior ocelli, becoming punctate reticulate posteriorly; gena punctate rugose; length of A3 1.1–1.2 × length of A2.

***Mesosoma*.** Cervical pronotal area densely punctate; dorsal pronotal area punctate rugulose; lateral pronotal area smooth dorsally, punctate rugulose ventrally; netrion densely finely punctate; notaulus shallow, foveolate; middle lobe of mesoscutum densely punctate, sculpture becoming denser anteriorly and posteriorly; lateral lobes of mesoscutum densely finely punctate throughout; mesoscutellum densely finely punctate throughout; metascutellum transverse, posterior margin slightly pointed medially, longitudinally carinate; propodeum continuous medially, not divided into two separated lobes, posterior margin narrowly notched medially, each side with several irregular longitudinal carinae medially, otherwise punctate rugose, covered by dense, recumbent, white setae; upper mesepisternum with a row of somewhat robust longitudinal carinae below subalar pit; lower mesepisternum variably smooth to punctate rugulose; mesopleural depression smooth; metapleuron punctate rugose throughout.

***Legs*.** Slender; hind femur weakly swollen, 3.60–3.80 × as long as its maximum width; hind tibia without spines over outer surface; hind basitarsus 9.60–10.20 × as long as its maximum width.

***Wings*.** Apex of fore wing extending from as far as middle of T5; R 1.56–1.67 × as long as r-rs, R1 1.63–1.70 × length of R.

***Metasoma*.** Posterior margin of transverse sulcus on T2 strongly convex; sublateral tergal carinae well developed on T1–T2, weakly developed on anterior half of T3; T1 densely longitudinally striate, with punctate rugulose sculpture in interstices anteriorly, punctate rugulose laterally; T2–T4 densely longitudinally striate with numerous large delicate punctures in interstices; T5–T6 densely punctate; length of T3 1.35–1.40 × length of T6; T5 distinctly wider than long; S2–S4 densely longitudinally striate, with delicate punctures in interstices; S5–S6 densely finely punctate; prominent longitudinal median carina absent on sternites.

**Male.** Differing from female as follows: body length 3.76 mm (*N* = 1); A1 yellow, the remainder of antenna dark brown to black; mesosoma dark brown to black dorsally, yellow laterally; T1–T4 densely longitudinally striate, with numerous delicate punctures in interstices; T5–T6 densely and finely punctate; T7 largely smooth except finely rugulose posterolaterally; T6 wider than long; length of T6 2.50 × length of T7; T7 transverse, apex truncate; length of T7 as long as S7; S7 granulate.

#### Distribution.

China (Xinjiang, Hebei, Inner Mongolia); Russia, Hungary.

#### Comments.

*Macroteleia variegata* is recorded here from China for the first time. We examined the holotypes of *M. variegata* and *M. ischtvani* and found no distinct differences between the two species except the trivial variations in colors and the relative length of metasomal tegites, which Kononova and Kozlov (2008) used heavily in the key to species of the Palearctic *Macroteleia*. Therefore, we here treat *M. ischtvani* as a synonym of *M. variegata*. We also examined a paratype of *M. elissa* Kozlov & Kononova, 1987 deposited in UASK that we believe is conspecific with *M. variegata*, but we cannot confirm if *M. elissa* should be treated as a synonym of *M. variegata* until we can examine the holoype of *M. elissa*. Color and size variations could be due to temperature or host egg size during the developmental stage of the parasitoids, which are quite commonly seen in Scelionidae. DNA barcoding could be useful in species delimitation for the species in such situations.

**Figures 25–30.**
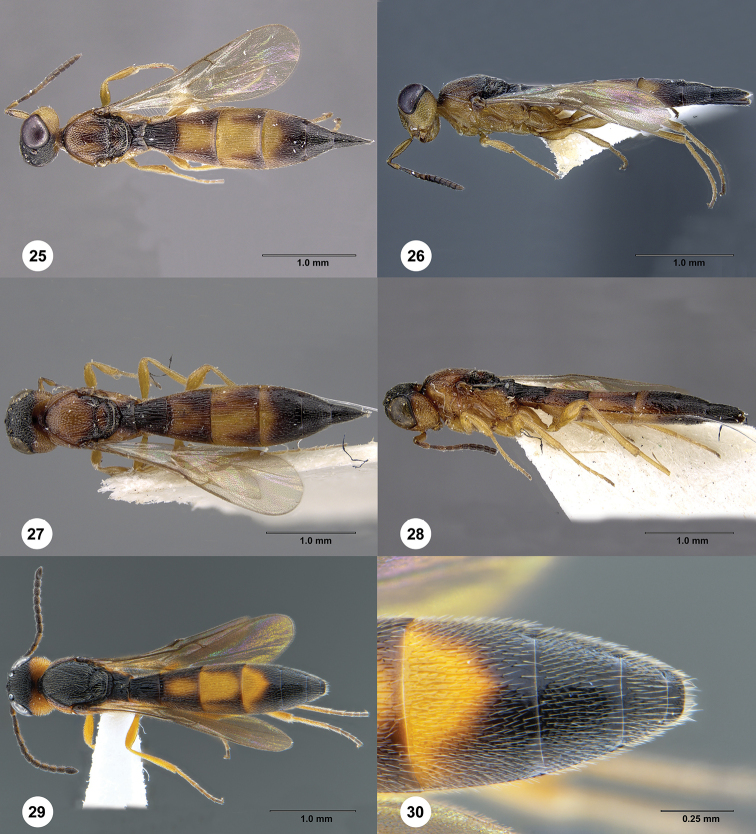
**25, 26***Macroteleia ischtvani* Kononova, holotype, female (UASK 0100): **25** dorsal habitus **26** lateral habitus **27, 28***Macroteleia variegata* Kozlov & Kononova, holotype, female (UASK 0104): **27** dorsal habitus **28** lateral habitus **29, 30***Macroteleia variegata* Kozlov & Kononova, male, (SCAU 3048584) **29** dorsal habitus **30** apex of metasoma, dorsal view.

**Figures 31–36.**
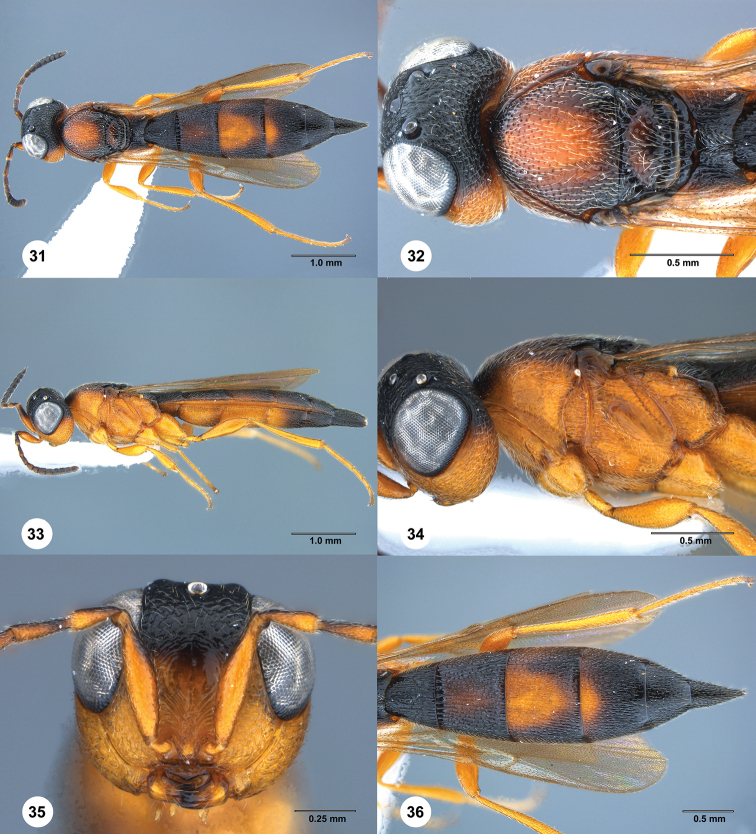
*Macroteleia variegata* Kozlov & Kononova, female, (SCAU 3041128) **31** dorsal habitus **32** head and mesosoma, dorsal view **33** lateral habitus **34** head and mesosoma, lateral view **35** head, anterior view **36** metasoma, dorsal view.

## Supplementary Material

XML Treatment for Macroteleia carinigena

XML Treatment for Macroteleia emarginata

XML Treatment for Macroteleia gracilis

XML Treatment for Macroteleia xui

XML Treatment for Macroteleia variegata
